# Supplementary material for: Sequential order dependent dark-exciton modulation in bi-layered TMD heterostructure
Source: Nat Commun. 2023 Sep 8;14:5548. doi: 10.1038/s41467-023-41047-6 (PMC10491585; doi:10.1038/s41467-023-41047-6)
Supplement: Supplementary file 1 — Supplementary Information [file 41467_2023_41047_MOESM1_ESM.pdf]

Supplementary information

Sequential order dependent dark-exciton modulation in bi-layered TMD heterostructure

Riya Sebait[1,2], Roberto Rosati[3], Seok Joon Yun[4], Krishna P Dhakal[1], Samuel Brem[3], Chandan Biswas[2], Alexander Puretzky[4], Ermin Malic[3✉], Young Hee Lee[1,2✉]

Affiliations:

[1]Deparment of Energy Science (DOES), Sungkyunkwan University, Suwon, 16419, Republic of Korea.

[2]Center for Integrated Nanostructure Physics (CINAP), Institute for Basic Science (IBS), Sungkyunkwan University, Suwon, 16419, Republic of Korea.

[3]Department of Physics, Philipps-Universität Marburg, Marburg, 35032, Germany.

[4]Center for Nanophase Materials Sciences (CNMS), Oak Ridge National Laboratory, Oak Ridge, 37830, United States.

✉e-mail: ermin.malic@physik.uni-marburg.de; leeyoung@skku.edu

Contents

1. **The procedure of vdW heterostructure preparation**
2. **Flake identification**
   i. Raman spectroscopy
   ii. Atomic force microscopy (AFM)
3. **Interface cleanness and strong coupling**
   i Fabrication method.
   ii A^2_{1g} peak at heterostructure
   iii Homogeneous topography image via AFM
   iv Modulation of A_{1g} intensity of WS_2 in heterostructure
4. **Identification of the emerging dark exciton peak**
5. **Deconvolution of PL spectra**
6. **Reproducibility of experimental results**
7. **Stacking angle measurement**
8. **Interlayer exciton**
9. **Substrate effect**
   i. Comparison of SiO_2 and h-BN substrate
   ii. Presence of S_Q peak on quartz and HfO_2 substrate
   iii. Exclusion of environmental effect
10. **Other TMD combinations**
11. **Effect of nano-bubbles**
12. **Homogeneous layer effect**
13. **Heterogeneous layer effect**
14. **Confirmation of Se_Q and S_Q peaks at different circumstances**
    i. Low-temperature and high vacuum PL measurements
    ii. Annealing effect
    iii. Doping effect
15. **Absorption measurement**
16. **Charge transfer and the related exciton dynamics**
17. **Mechanical cleaning *via* AFM tip**
18. **Scanning tunneling spectroscopy (STS) measurement**
19. **Laser power-dependent measurement of WS_2/WSe_2 on SiO_2**
20. **S_Q intensity mapping**
21. **Raman spectra at different positions**
22. **Schematic representation of compressive force**
23. **Theoretical calculations for comparing the K-Q to K-K excitons for W- and Mo-based materials**
24. **Independent of optical excitation path**
25. **References**

Note 1. The procedure of vdW heterostructure preparation

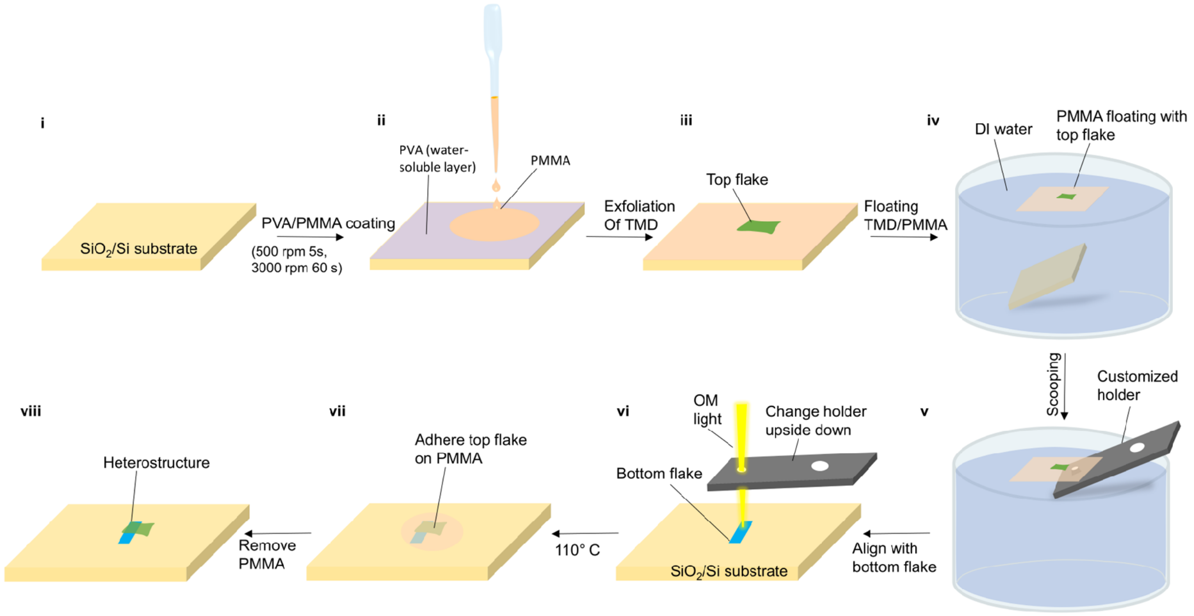

Fig. S1 | Schematic illustration of a stepwise fabrication process of TMDs hetero-bilayer via PMMA-assisted layer. Top flake preparation: exfoliation on PVA/PMMA substrate, bottom flake: direct exfoliation on the substrate Finally, the top flake is transferred onto the destinated bottom flake through align transfer method.

Note 2. Flake identification

i. Raman spectroscopy

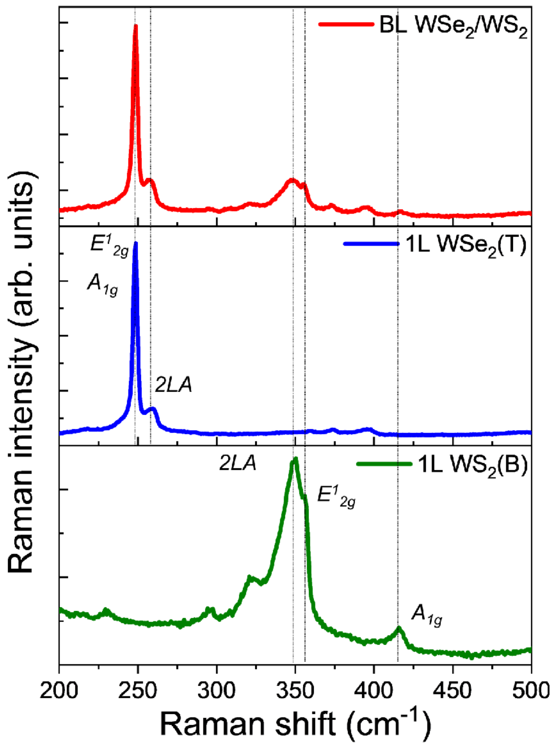

Fig. S2 | Raman spectra of each monolayer along with heterostructure region, identifying several Raman modes for A_{1g} and $E^1{}_{2g}$ for WS_2 and WSe_2 monolayer and the corresponding heterostructure.

ii. Atomic force microscopy (AFM)

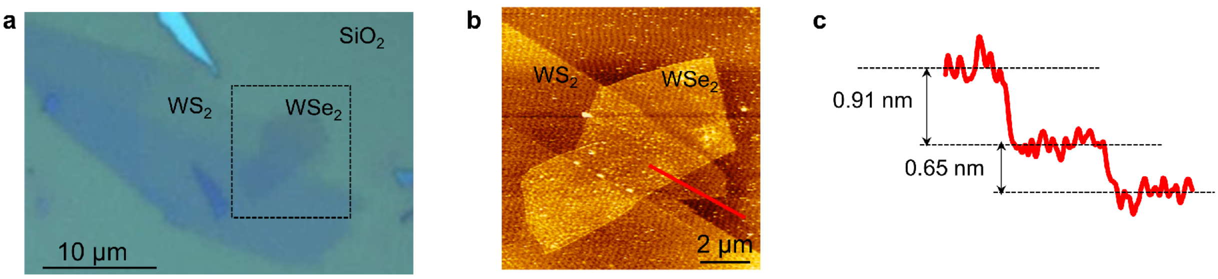

Fig. S3 | Height profile by atomic force microscopy (AFM). a, Optical image of WSe_2/WS_2 heterostructure. **b,** AFM image of the black-dotted box region as shown in **a**. **c,** Height profile across the line cut (red color in **b**), which confirms the presence of respective monolayers WSe_2 and WS_2.

Note 3. Interface cleanness and strong coupling

i. Fabrication method.

Dark exciton peaks (S_Q or Se_Q) were exclusively observed from the clean interface. To confirm this, we fabricated one heterostructure (Supplementary Fig. S4a) as described in the method section, where the interface remained inert during transfer. Meanwhile, another heterostructure, where the bottom layer was transferred with polypropylene carbonate (PPC) to the target substrate and then cleaned the polymer by the conventional acetone-IPA-ethanol cleaning process, followed by the top layer transferred on top of it. Even though polymers were cleaned, still some contamination remained. As a consequence, we did not find a clear signature of a Q-band-related dark exciton peak (S_Q) in this case (Supplementary Fig. S4b).

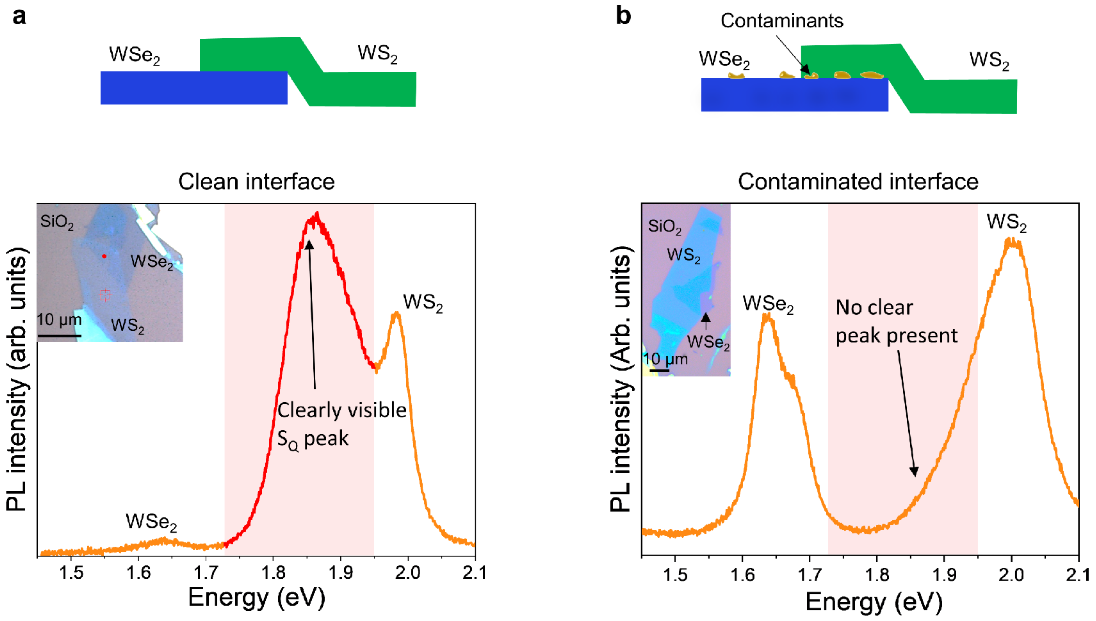

Fig. S4 | Interface cleanness. a, Schematic representation of WS_2/WSe_2 heterostructure. A clear S_Q peak was observed from the clean interface obtained by our transfer method. **b,** Schematic representation of contaminated WS_2/WSe_2 heterostructure and the S_Q peak was not visible in this case.

ii. A^2_{1g} peak at heterostructure

A^2_{1g} Raman mode becomes active in homo-bilayer or thicker layers and remains inactive in monolayer. It is noteworthy that, active A^2_{1g} mode of the WSe_2 layer was clearly observed in a strongly coupled heterostructure (Supplementary Fig. S5a), whereas it became less intense in a weakly coupled heterostructure (Supplementary Fig. S5b). Previously, a similar result was reported for MoS_2/WSe_2 heterostructure, where A^2_{1g} mode was inactive before annealing and A^2_{1g} mode become active after annealing (at 300 °C) due to strong coupling between the layers.[1] However, in our case, we observed A^2_{1g} (309 cm^{-1}) without annealing the sample, which again confirms the strongly coupled cleaned interface of our heterostructure.

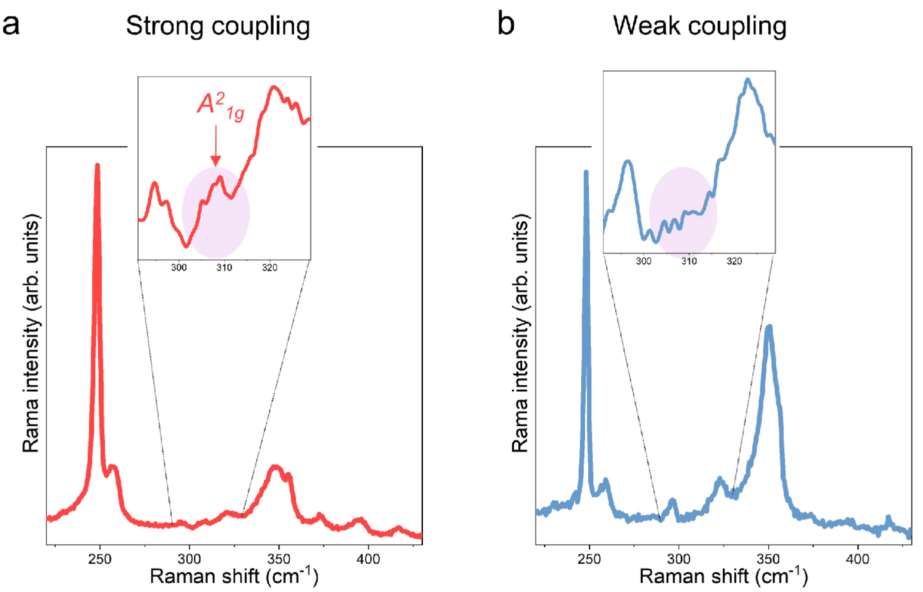

Fig. S5 | Activation of A^2_{1g} mode at strongly coupled hetero-bilayer. a, Raman spectrum of WSe_2-WS_2 hetero-bilayer. A^2_{1g} Raman mode (309 cm^{-1}) becomes active at a strongly coupled heterostructure indicated by an arrow (inset). **b,** In a weakly coupled WSe_2-WS_2 hetero-bilayer case, A^2_{1g} peak remains inactive.

iii. Homogeneous topography image via AFM

Homogeneous AFM topography image of the heterostructure region (Supplementary Fig. S6a,b) with corresponding low height fluctuation with distance (Supplementary Fig. S6c) represents a bubbles/contaminations-free clean interface. Meanwhile, the trap bubbles/contaminations are rich in a weakly coupled interface, which shows large root mean square surface roughness (Supplementary Fig. S6d,e) with large fluctuations of profile roughness (Supplementary Fig. S6f).

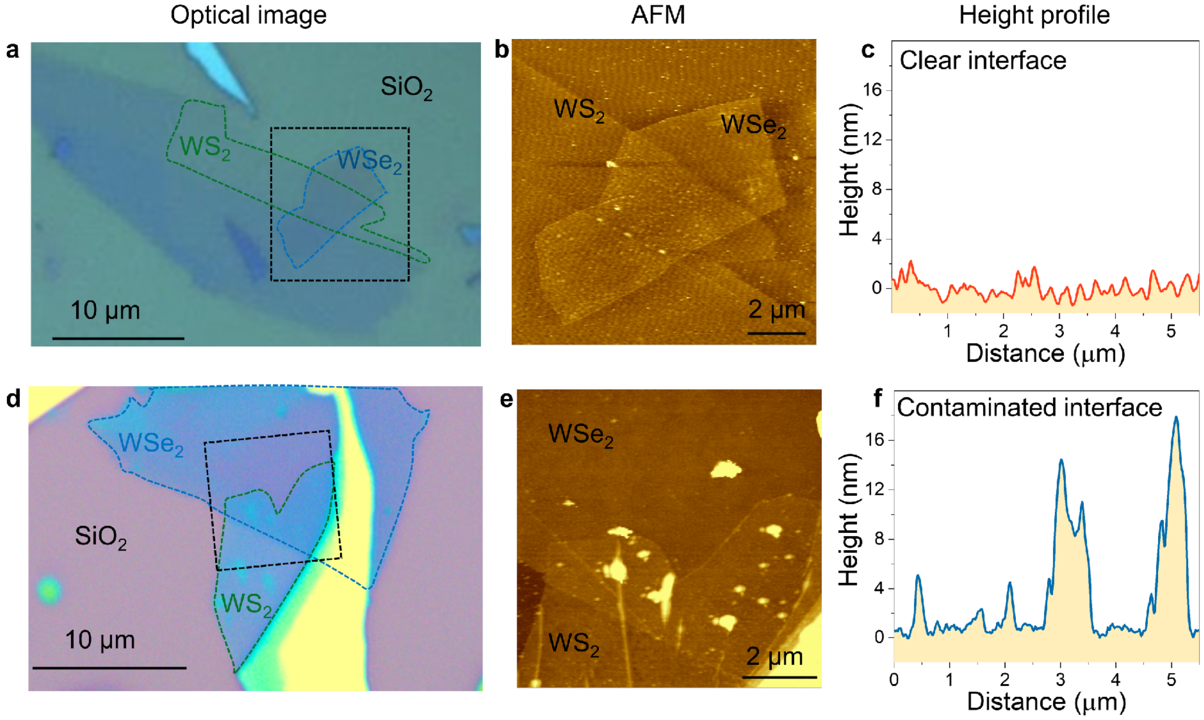

Fig. S6 | Homogeneous topography image via AFM. Optical images **a** and **d**, AFM images **b** and **e** as well as height profile **c** and **f** of WSe_2-WS_2 hetero-bilayer for strong and weakly coupled heterostructures, respectively. Bright yellow dot spots in **e** represent the air trap bubbles/contaminants.

iv. Modulation of A_{1g} intensity of WS_2 in heterostructure

The relative intensity of A_{1g} mode is always smaller in monolayer and increases with layer numbers as observed previously.[2,3] In our investigation we also observed similar properties in monolayer and bilayer WS_2 as shown in Supplementary Fig. S7a. Now, we also observed highly intense A_{1g} peak at hetero-bilayer (such as $WS_2/MoSe_2$ and WSe_2/WS_2) heterostructure as well, similar to homo-bilayer WS_2 (Supplementary Fig. S7b,c), compared to individual monolayer WS_2. Furthermore, to clarify it in detail, we took the intensity ratio of the bilayer with monolayer for three significant peaks (2LA, E^1_{2g}, and A_{1g}) (Supplementary Fig. S7a, right), which shows a high A_{1g} intensity, compared to other modes. This property is further followed by heterogenous bilayer cases as well (Supplementary Fig. S7b,c right), which again confirms the strong coupling between the hetero-bilayer.

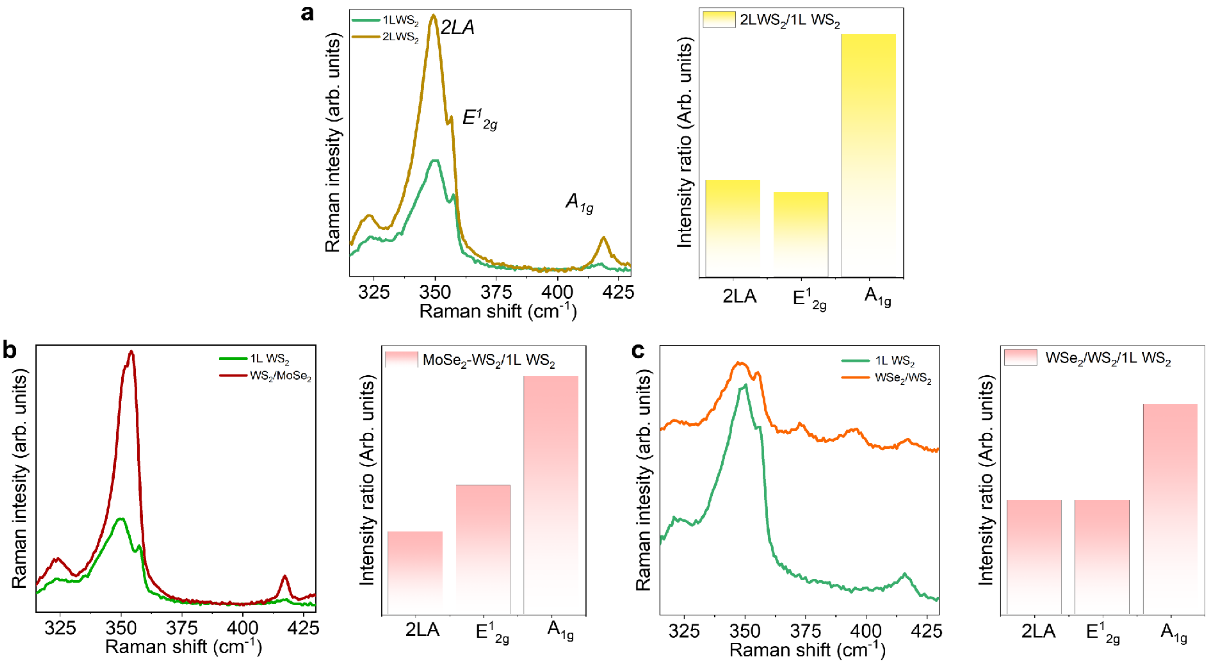

Fig. S7 | A_{1g} peak intensity at hetero-bilayer. a, In WS_2 bilayer, the relative intensity of A_{1g} peak is higher compared to the monolayer. **b,c,** Similar high intensity of the A_{1g} peak was observed in $WS_2/MoSe_2$ and WSe_2/WS_2 heterostructure.

Note 4. Identification of the emerging dark exciton peak

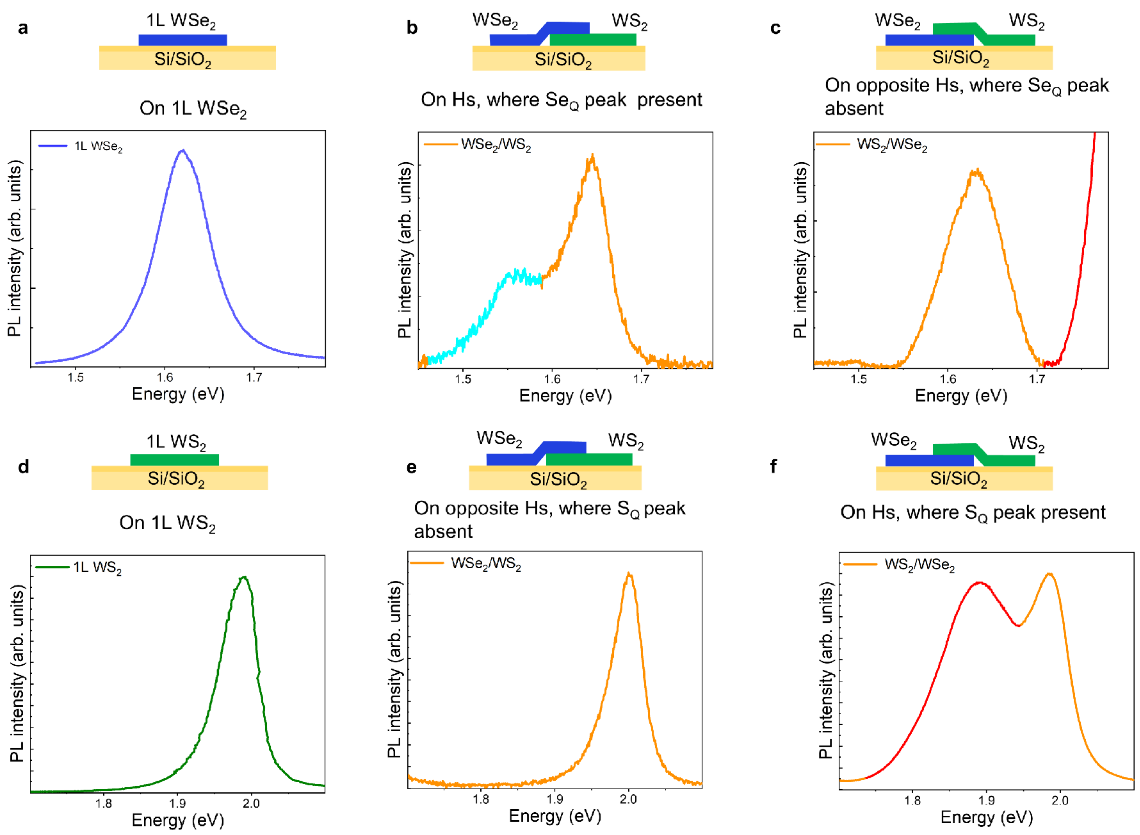

Fig. S8 | Presence of Se_Q and S_Q peak. a, PL spectrum of monolayer WSe_2. **b,** PL spectrum at top-WSe_2/bottom-WS_2 heterostructure, where clear Se_Q emerges at an energy of 1.56 eV. **c,** PL spectrum at top-WS_2/bottom-WSe_2 heterostructure, where the Se_Q peak is absent. **d,** PL spectrum of monolayer WS_2. **e,** PL spectrum at the top-WSe_2/bottom-WS_2 heterostructure, where Se_Q is absent. **f,** PL spectrum at the top-WS_2/bottom-WSe_2 heterostructure, where the prominent S_Q peak is present.

Note 5. Deconvolution of PL spectra

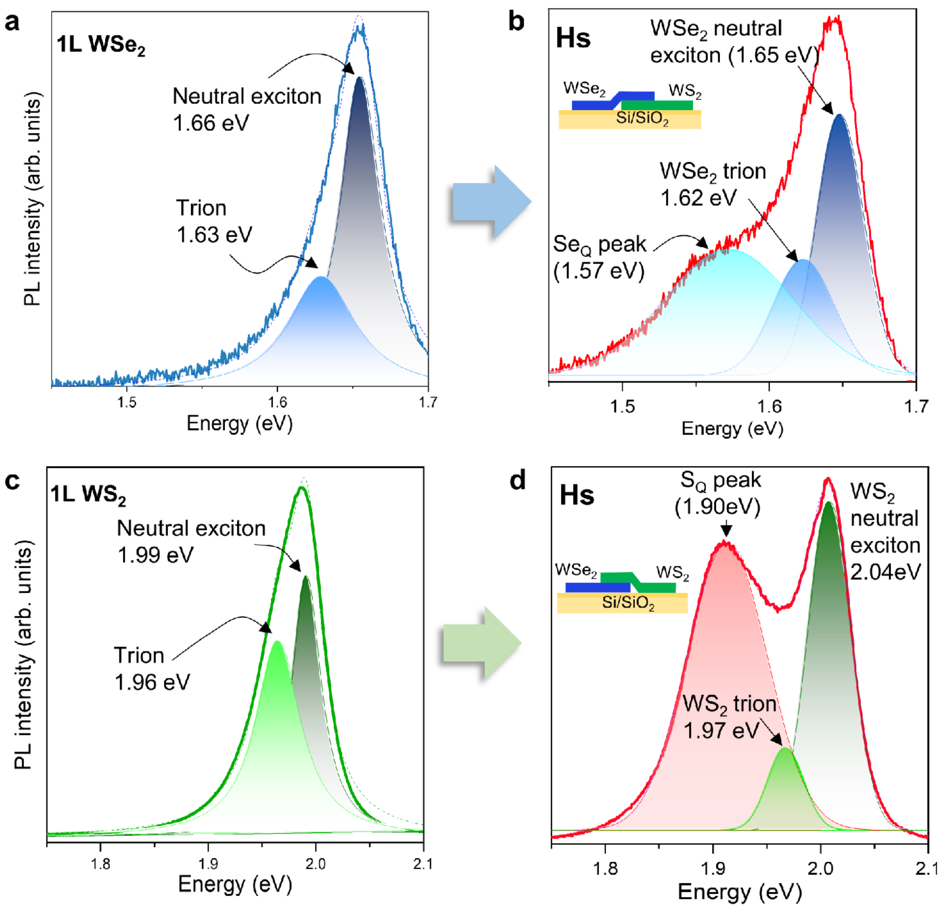

Fig. S9 | Deconvolution of the PL spectra. a, Deconvoluted PL spectrum of monolayer WSe_2 region, fitted with neutral exciton (1.66 eV) and trion (1.63 eV). **b,** PL spectrum at WSe_2/WS_2 heterostructure region fitted by three peaks, along with WSe_2 neutral exciton (1.65 eV) and trion (1.62 eV), dark exciton (Se_Q) emerges at 1.57 eV. **c,** Deconvoluted PL spectrum of monolayer WS_2 region, fitted with neutral exciton (1.99 eV) and trion (1.96 eV). **d,** Unusual peak (S_Q) emerges at 1.90 eV exclusively on the top WS_2 layer of heterostructure region along with WS_2 neutral exciton (2.04 eV) and trion (1.97 eV).

Note 6. Reproducibility of experimental results

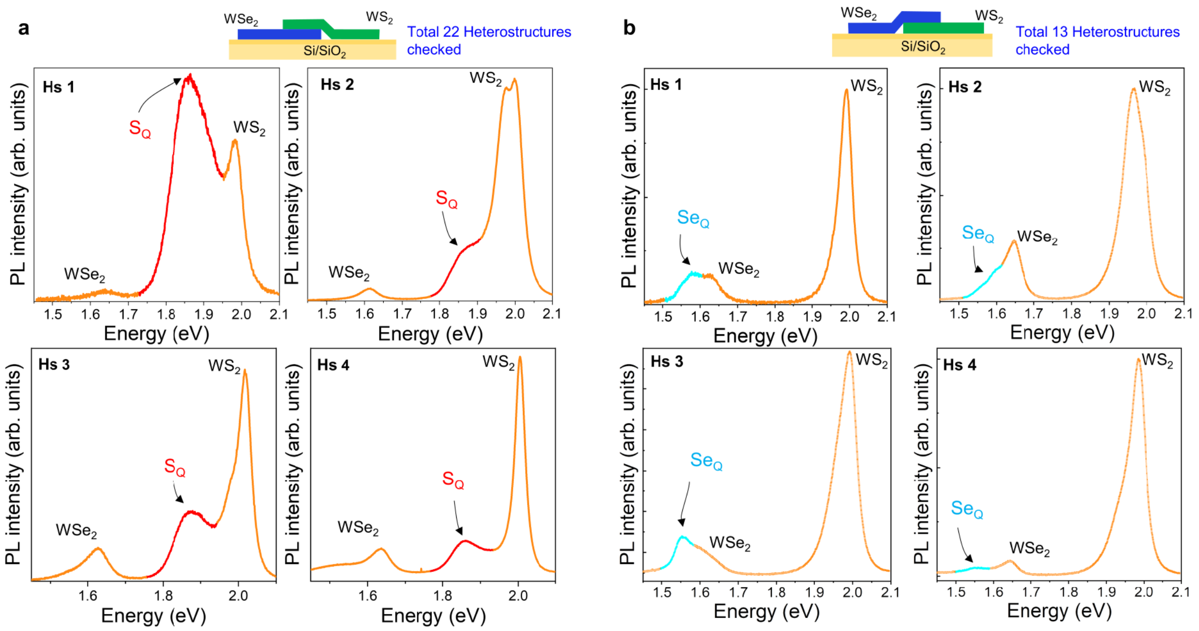

Fig. S10 | Data reproducibility. a,b, Represents the PL spectra of WS_2/WSe_2 with 22 samples and inverted WSe_2/WS_2 stacking sequence with 13 samples.

Note 7. Stacking angle measurements

We performed second harmonic generation (SHG) measurements on seven different samples to determine the stacking angle between the layers (Table S1), where we observed the indirect K-Q peaks, as illustrated in Supplementary Fig. 11. Based on the observations, we conclude that our newly observed K-Q intralayer peak is independent of the stacking angle, making it more robust compared to moiré or interlayer excitons.

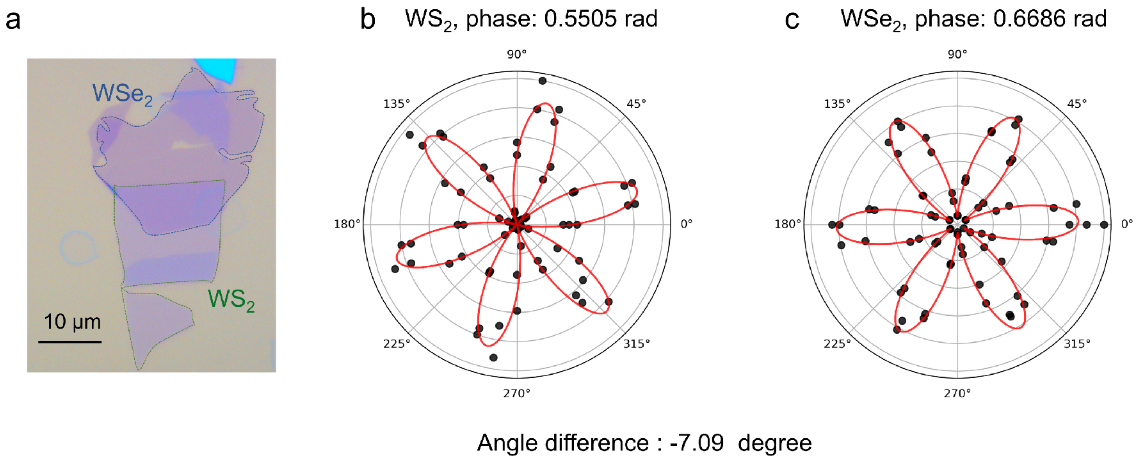

Fig. S11 | Twist-angle measurements using second-harmonic generation (SHG). a, Optical image of WS_2/WSe_2 heterobilayer **b,c,** Polarized SHG intensity polar diagram as a function of incident field polarization angle for WS_2 and WSe_2 respectively. The black dots correspond to experimental data and the red line is the fitting, which reveals the stacking angle between two layers is -7.09°.

Table S1 | Stacking angle measured via second harmonic generation (SHG).

Sample name	Stacking sequence (top/bottom) layer	Stacking angle (degree)
RD13	WS_2/WSe_2	16.36
RD14	WSe_2/WS_2	15.14
RD26	WS_2/WSe_2	8.48
RD51	WS_2/WSe_2	24.53
RD52	WS_2/WSe_2	24.31
RD64	WS_2/WSe_2	-7.09
RD73	WS_2/WSe_2	35.34

Note 8. Interlayer exciton

We rarely observed the interlayer exciton (ILE). For example, in $MoSe_2/WSe_2$ heterostructure, a weak ILE peak was observed at an energy of1.35 eV at room temperature (Supplementary Fig. S12), which is well matched with previously reported results.[4–6] However, in most of the cases, we have not observed any signature of interlayer excitons. The reason behind this is listed below:

A) In general, interlayer exciton requires specific angle alignment between the layers as observed previously.[7–11] However, in our case, heterostructures are not fabricated with specific angle alignment.

B) Interlayer excitons are most prominent at low temperatures.[5,7–9,12,13] Nevertheless, in this work, all the experiments have been conducted at room temperature.

C) Interlayer exciton generally appears, i.e., at the energy of 1.35-1.45 eV for WSe_2-WS_2 heterostructure as reported previously,[7–9,13]which is beyond the scope of this work.

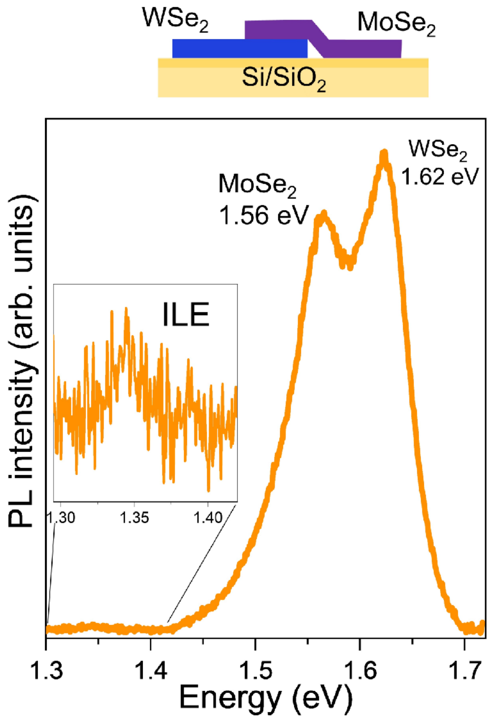

Fig. S12 | Interlayer exciton. Interlayer exciton (ILE) peak appears at an energy of 1.35 eV in $MoSe_2/WSe_2$ heterostructure at room temperature.

Note 9. Substrate effect

i. Comparison of SiO_2 and h-BN substrate

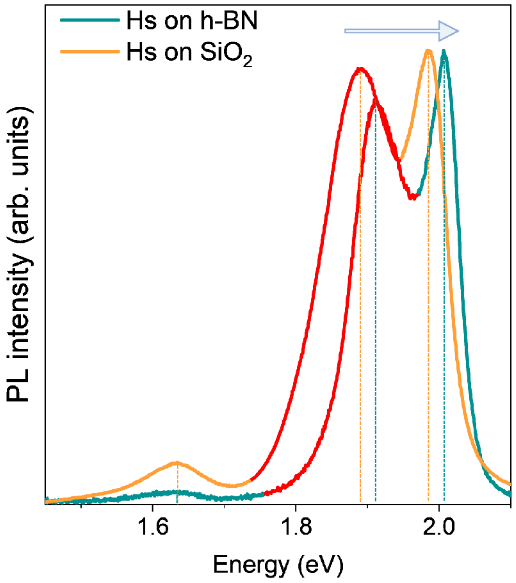

Fig. S13 | Upshifted PL peak of WS_2 in WS_2/WSe_2 heterostructure on h-BN substrate compared to SiO_2 substrate due to reduced charge screening.

ii. Presence of S_Q peak on quartz and HfO_2 substrate

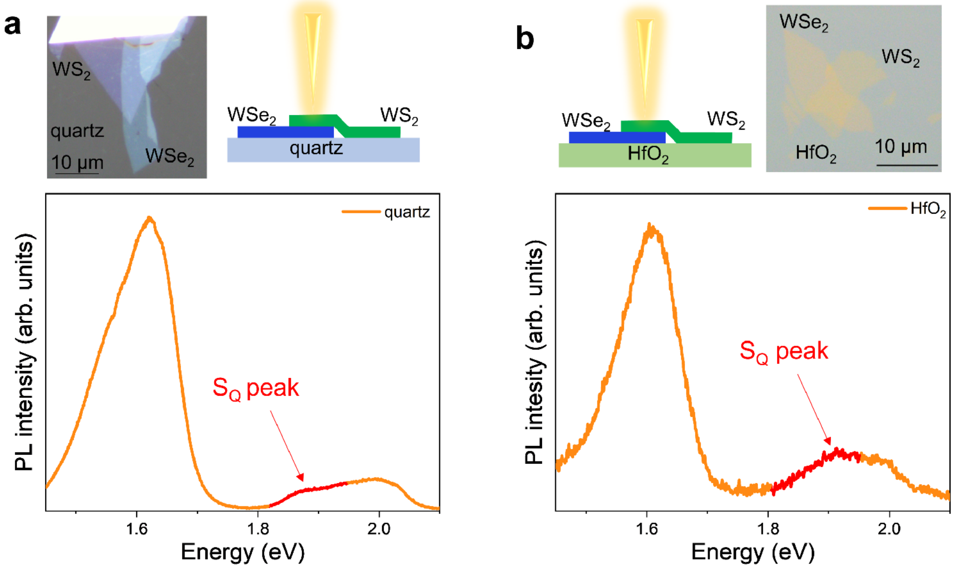

Fig. S14 | Dielectric effect on different substrates. a,b, PL spectra of WS_2/WSe_2 heterostructure on respective quartz and HfO_2. S_Q peak (red) on top WS_2 layer is present on both substrates.

iii. Exclusion of environmental effect

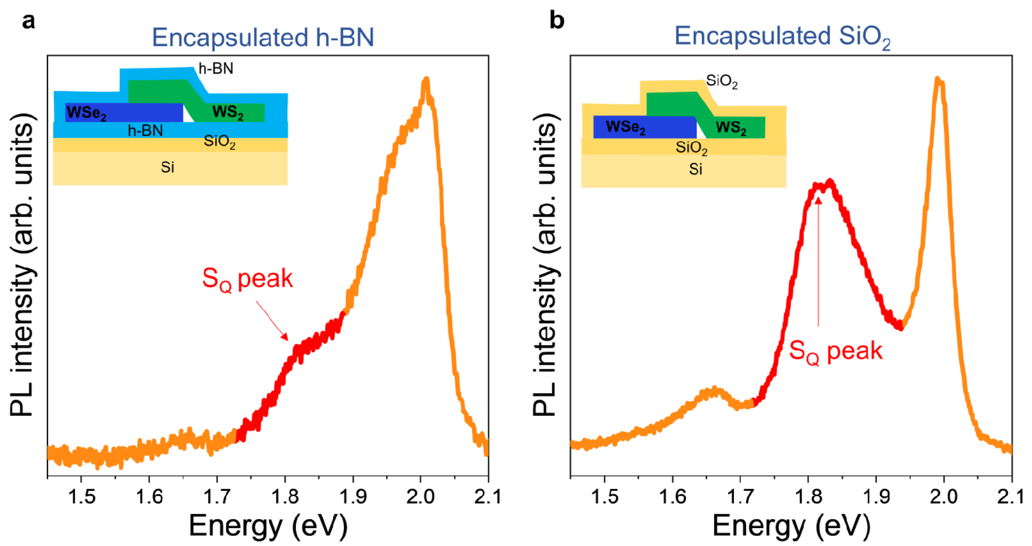

Fig. S15 | Environmental effect. a,b, PL measurements of WS_2/WSe_2 heterostructures on encapsulated h-BN and SiO_2. S_Q (red) peaks still appear, except for a small energy change due to band renormalization.

Note 10. Other TMD combinations

We further investigated several hetero-bilayers with other types of TMDs, for example, MoS_2-WSe_2, $MoSe_2$-WSe_2 and MoS_2-WS_2 (Supplementary Fig. S16) heterostructures. In top-WSe_2/bottom-MoS_2 hetero-bilayer case, we have observed a similar Se_Q peak as earlier, due to the Q band of the WSe_2 layer being downshifted at the hetero-bilayer region (Supplementary Fig. S16a, left). On the other hand, top-MoS_2/bottom-WSe_2 hetero-bilayer does not show any appreciable peak. This could be due to the Q-band of MoS_2 being located far away from the K band,[14] becoming difficult to renormalize to form the hybrid K-Q state or could be inferred to the overlap of the energy range with the WSe_2 band gap (Supplementary Fig. S16a, right).

In the WSe_2-$MoSe_2$ hetero-bilayer case, we have not observed any kind of dark exciton-related PL peak (Supplementary Fig. S16b). This can be explained with WSe_2 as the top layer that the Se_Q generally emerges at lower energy than WSe_2 A-exciton and thus this energy is again also overlapped with $MoSe_2$ A-exciton (Supplementary Fig. S16b, left). Similarly, $MoSe_2$ as a top layer is expected to have an emerging dark exciton at higher energy than $MoSe_2$ A-exciton in analogy with what was observed for $MoSe_2$/WS_2 hetero-bilayer (Fig. 1f). Similarly, the dark exciton peak is likely overlapped with WSe_2 A-exciton peak in this case (Supplementary Fig. S16b, right). Nevertheless, we have not observed such a peak for both stacking sequences, which can be considered as an exception since other tested bilayer heterostructures generally revealed the clear emergence of dark exciton peaks on the top layer.

In WS_2(top)/MoS_2(bottom) case, the notable S_Q peak has been observed as WS_2 as the top layer with an energy of 1.89 eV, similar to other TMDs combinations (red color peak in Supplementary Fig. 16c, left). On the other hand, in MoS_2(top)/WS_2(bottom) heterobilayer case, we have not observed any dark exciton-related PL peak (Supplementary Fig. S16c, right). This can be explained with MoS_2 being in the top layer where the K-Q peak for Mo-based material then emerges at higher energy than MoS_2 A-exciton (as we observed for the $MoSe_2$ case: Fig. 1f) and this K-Q energy overlaps with inlayer WS_2 exciton. Another reason could be the energy of the Q-band, which is located far away from the K-band,[14] subsequently incapable of band renormalization for K-Q excitons. The B-exciton of MoS_2 is also indistinguishable for the same overlapping energy with inlayer WS_2 exciton. We note that a similar PL peak to our S_Q peak has been observed previously in WS_2/MoS_2 heterobilayer.[15]

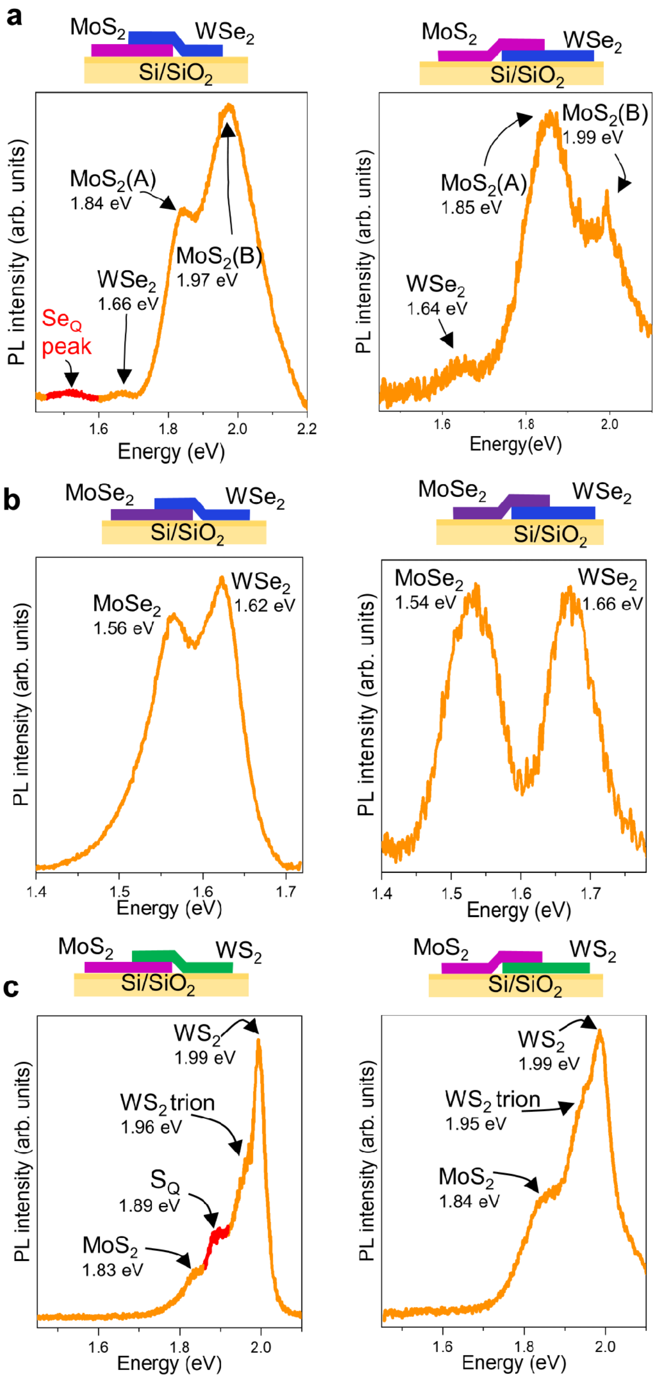

Fig. S16| Stacking sequence effect with other TMD combinations. a, PL spectra of WSe_2/MoS_2 and inverted MoS_2/WSe_2 heterostructure. Se_Q still emerges on the WSe_2 layer of WSe_2/MoS_2 heterostructure, whereas the S_Q peak in MoS_2 is rather ambiguous. **b,** PL spectrum of $MoSe_2/WSe_2$ and $WSe_2/MoSe_2$ heterostructure. The dark exciton peak was not visible due to overlaps with the bandgap. **c,** PL spectra of WS_2/MoS_2 and inverted MoS_2/WS_2 heterostructure. Se_Q still emerges on the WS_2 layer of WS_2/MoS_2 heterostructure, whereas the S_Q peak in MoS_2 is indistinguishable.

Note 11. Effect of nano-bubbles

To distinguish between the localized excitons originating from nano-bubbles[16] and the K-Q exciton in the heterostructure, we performed PL mapping on WS_2/$MoSe_2$ heterobilayer, which contains several bubbles (indicated by the arrow in Supplementary Fig. S17a,b) at the interface. We found that on top of the nanobubble, the WS_2 inlayer peak underwent a red shift due to strain, while the $MoSe_2$ peak remained unaffected (Supplementary Fig. S17c). Conversely, on the flat surface, we observed a prominent and highly intense S_Q peak. Additionally, the relative intensity of the bottom $MoSe_2$ layer increased at the flat surface, indicating strong coupling between the layers, which is deemed necessary for a high S_Q peak. Supplementary Fig. S17d,e, represents the deconvolution of each spectrum from the flat and nanobubble region, confirming the presence of a strong S_Q peak on the bubble-free flat surface. This finding again supports the conclusion that the S_Q peak is not originated from an interfacial bubble but rather arises due to strong interlayer coupling between the layers.

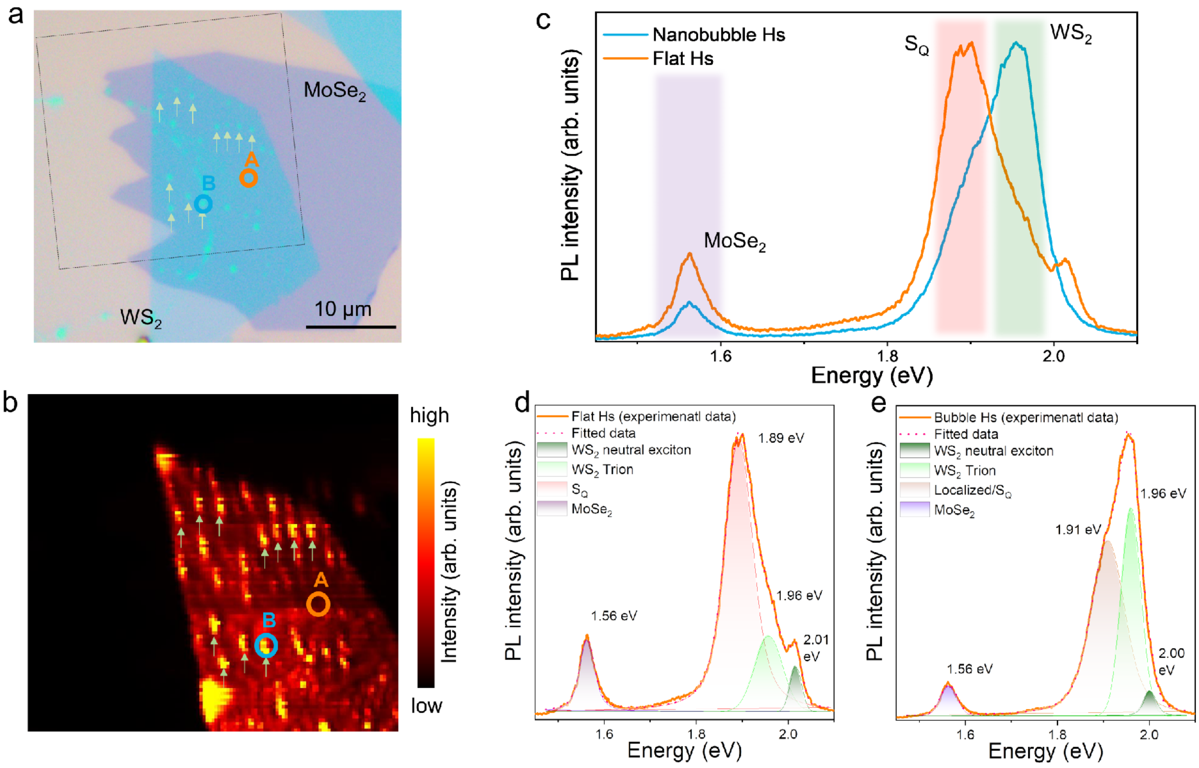

Fig. S17 | Effect of nano-bubble. a, Optical image of WS_2/$MoSe_2$ heterobilayer. Nano-bubbles are indicated by arrows. **b,** Spatial PL map of WS_2/$MoSe_2$ heterobilayer, revealing that bright spots are localized in spatially discrete regions corresponding to the presence of nano-bubbles. **c,** Comparison of PL spectra collected from flat (labeled as A: orange) and nano-bubble region (labeled as B: blue). **d,e,** Deconvolution of the spectrum from the flat and nano-bubble region.

Note 12. Homogeneous layer effect

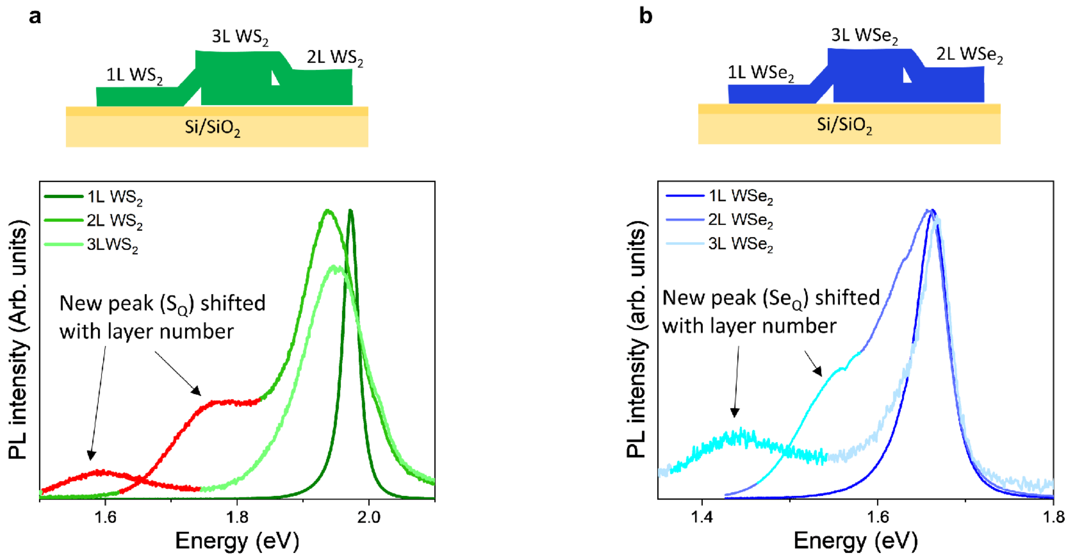

Fig. S18 | Layer-dependent PL measurement. a,b, WS_2 and WSe_2 layer-dependent PL measurement. Monolayer does not have an extra peak where bi or tri-layer has an emerging peak below their corresponding A-exciton energy (red and cyan color, respectively).

Note 13. Heterogeneous layer effect

We have investigated the heterostructures with different numbers of layers (Supplementary Fig. S19), which interestingly follow similar stacking sequence behavior. Supplementary Fig. S19a represents the PL measurement at 1L-WS_2/2L-WSe_2 heterostructure. S_Q (red color peak) is present at WS_2/WSe_2 heterostructure interface. Se_Q is also present in this system as well, as this heterostructure consists of bilayer WSe_2. Other than the nearest neighbor such as X^T, S_Q^T or Se_Q^T peaks represents corresponding trion peaks. Supplementary Fig. S19b represents the PL at 2L-WSe_2/1L-WS_2 heterostructure. Here S_Q peak is absent, as WS_2 is situated at the bottom. However, the Se_Q peak is still observed due to the top-WSe_2 layer in the heterostructure as well as the bi-layer of WSe_2. In this case, their energy is very close to each other. We cannot distinguish these two types of Se_Q peaks. Again, we measured the PL at 2L-WS_2/1L-WSe_2 (Supplementary Fig. S19c). In this case, we have two S_Q peaks, one appearing due to the WS_2/WSe_2 interface (S_{Q1}: red) and another emerging due to the bilayer nature of WS_2 (S_{Q2}: yellow). Since these peaks are well separated, we can detect them individually. The violet color peak (S_Q^T) near the red-S_Q peak is ascribed to the trion state of S_Q. Further PL spectrum measured at an even number of hetero-bilayer such as 2L-WS_2/2L-WSe_2 (Supplementary Fig. S19d) and shows one Se_Q peak and two S_Q peaks; the first S_Q peak (S_{Q1}: red) emerges at WS_2/WSe_2 interface and second S_Q (S_{Q2}: yellow color peak) originates from the bilayer of WS_2. Similar to these experiments, we also measured the PL spectrum from the 2L-WS_2/3L-WSe_2 heterostructure (Supplementary Fig. S19e), where only the

S_{Q1} peak is visible at WS_2/WSe_2 interface. However, the S_{Q2} peak becomes ambiguous because of the low signal-to-noise ratio. One Se_Q peak is also visible due to bottom 3L-WSe_2. We note that this Se_Q peak is downshifted compared to previous case due to its tri-layer nature. From all these various layers-dependent PL measurements we confirm that S_Q or Se_Q peak does not depend on inversion symmetry or mirror symmetry of the system as these peaks are observed for both even and odd layer numbers of the heterostructure.

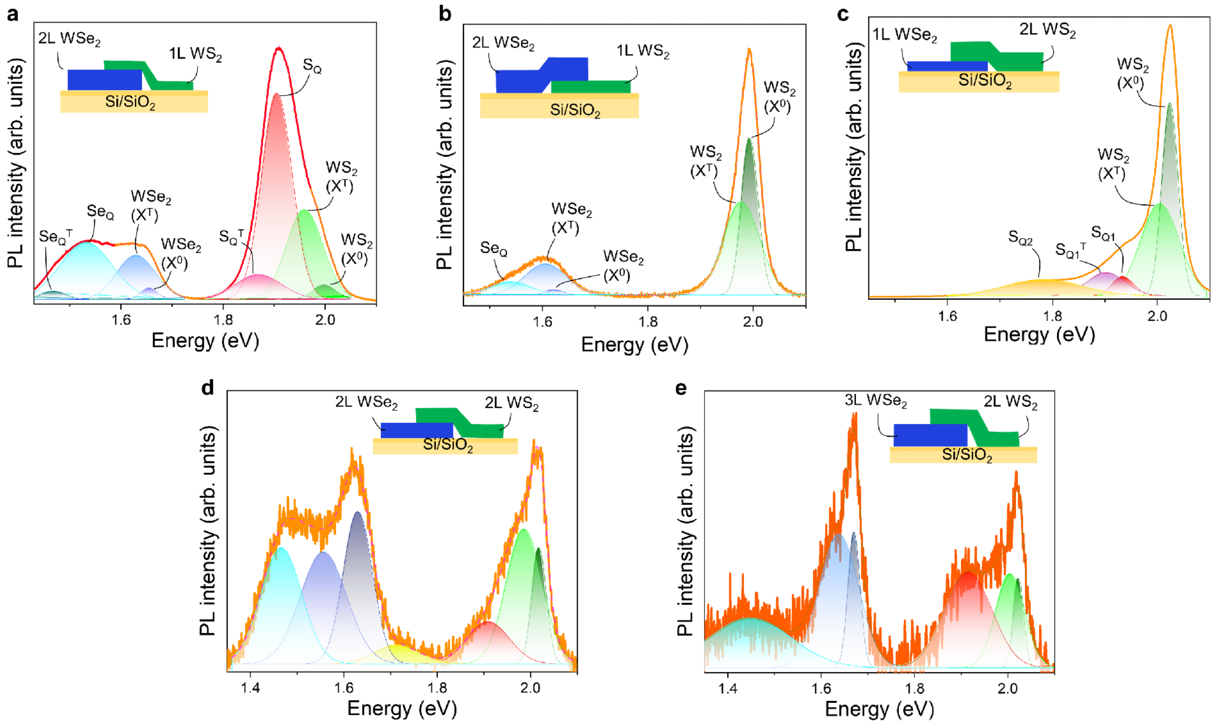

Fig. S19 | Various layer-dependent PL measurements for inversion symmetry effect test in heterostructures. **a-e,** WS_2-WSe_2 various layer-dependent PL spectrums with different stacking sequences.

Note 14. Confirmation of Se_Q and S_Q peaks at different circumstances

i. Low-temperature and high-vacuum PL measurements

We further confirmed the similar dark exciton peak S_Q or Se_Q depending on the corresponding stacking sequence at low temperature (77K) and high vacuum (~10^{-6} Torr). The green and blue dotted lines represent the individual monolayer PL peak of WS_2 and WSe_2, respectively. For each case, the PL peak was slightly upshifted compared to the room temperature measurement similar to previous studies.[12,17] In the WSe_2/WS_2 stacking sequence, we again observed the Se_Q peak present even at low temperatures (Supplementary Fig. S20a). There is another PL peak observed with even lower energy than the Se_Q peak at low temperatures that could be related to the associated trion state. Similarly, in the WS_2/WSe_2 stacking sequence, S_Q still appeared at low temperatures as well (Supplementary Fig. S20b).

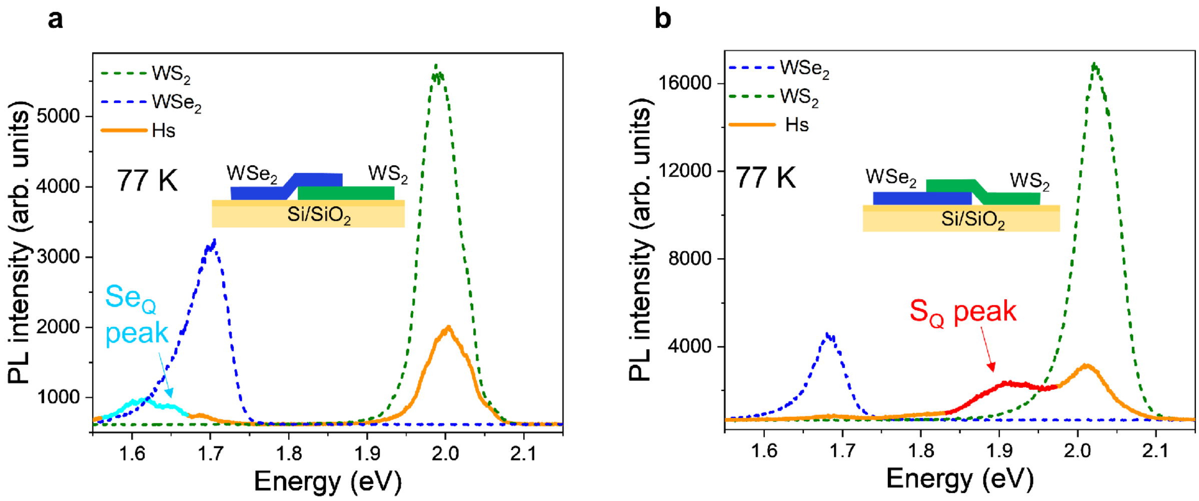

Fig. S20 | Low-temperature PL measurement. a,b, PL measurement at low temperature (77K) and high vacuum (~10^{-6} Torr) of WSe_2-WS_2 heterostructure with opposite stacking (**a** and **b**). The corresponding peaks (Se_Q and S_Q) are present in both cases.

ii. Annealing effect

We annealed our hetero-bilayer samples at 250° C for 12 hours in helium (He) environment to improve the interlayer distance as well as to remove the polymeric residues.[12,15] Therefore, we measured PL spectra after fabricating the heterostructure and before annealing the sample. We annealed the heterostructure and again measured the PL of the same sample (Supplementary Fig. S21). We observed that before and after annealing, the same S_Q (Se_Q) exists for the WS_2/WSe_2 (WS_2/WSe_2) stacking sequence with a slight peak shift. From these results, we confirm that these peaks originate from the intrinsic material properties and are not related to any defect or contamination-generated photoluminescence.

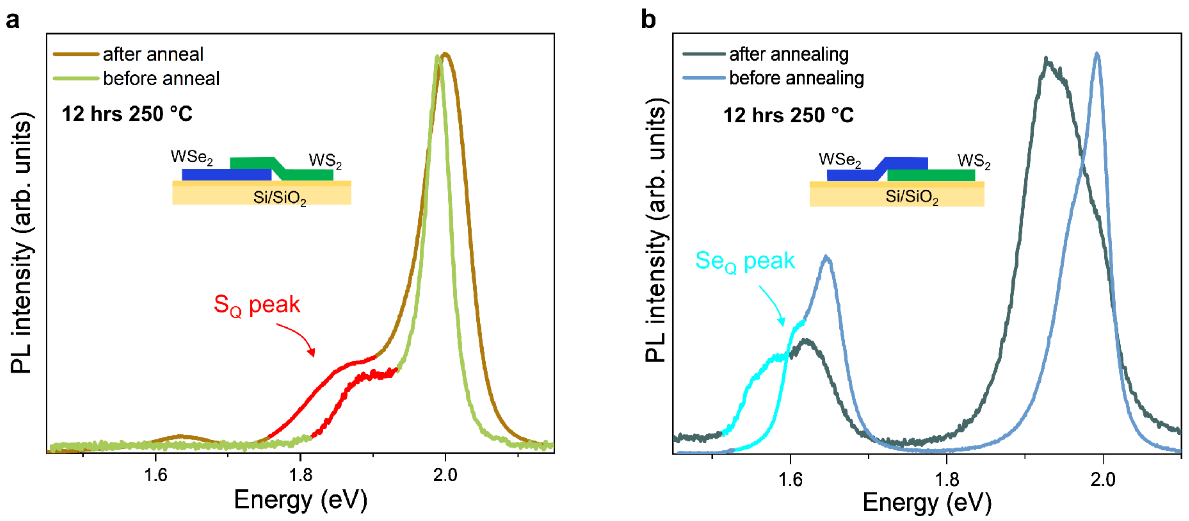

Fig. S21 | PL characterization of thermal annealing influence. a,b, Before and after annealing, both S_Q (red color peaks) and Se_Q (cyan color peaks) were present in WS_2/WSe_2 and WSe_2/WS_2 hetero-bilayer, respectively.

iii. Doping effect

We have increased the number of carriers by intrinsic Fermi level doping and again checked the stacking sequential properties of the WS_2-WSe_2 hetero-bilayer. In this case, we have used rhenium-doped WS_2 (Re-WS_2) for increasing more n-type carriers and niobium-doped WSe_2 (Nb-WSe_2) to increase the p-type carrier. Afterward, we fabricated Re-WS_2/Nb-WSe_2 with opposite stacking (Nb-WSe_2/Re-WS_2) and measured the PL for each case. We observed similar peaks (S_Q or Se_Q) on dopped material (Supplementary Fig. S22) as well for their corresponding stacking sequences analogous to undoped hetero-bilayers.

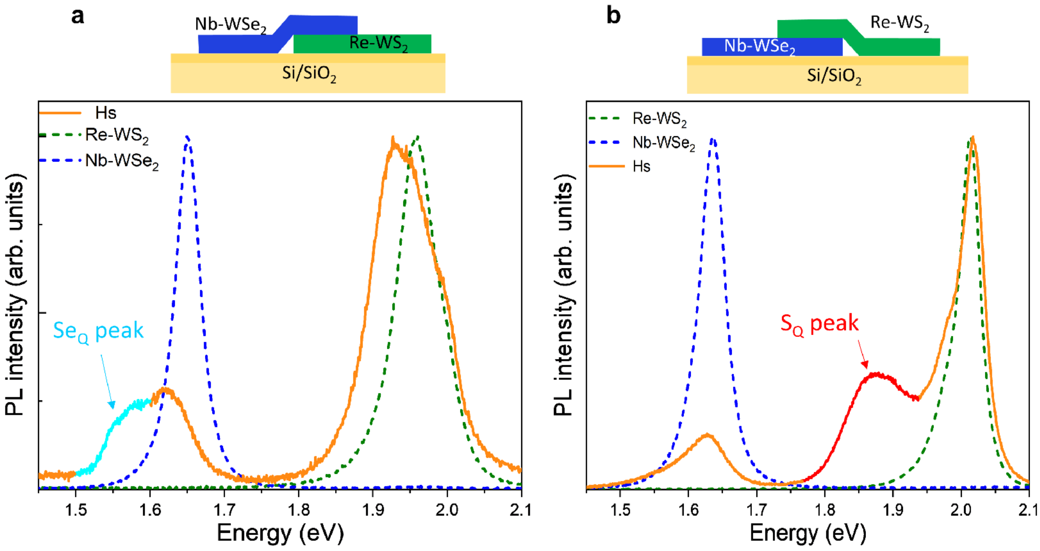

Fig. S22 | PL measurement of doping effect. a, PL spectra of the individual monolayer of Nb-WSe_2 and Re-WS_2 (blue and green dotted lines, respectively) along with the Nb-WSe_2/Re-WS_2 heterostructure region (yellow solid line). Se_Q (cyan color peak) is still present in the doped sample. **b,** Similar to **a** for opposite stacking (Re-WS_2/Nb-WSe_2) measurement, where S_Q peak (red color peak) persists analogous to undoped sample.

Note 15. Absorption measurements

Measuring the indirect bandgap through the absorption spectrum in TMD heterostructures poses a challenging task due to the involvement of both direct and indirect bandgaps, along with the band renormalization effect. This is well contrasted with PL, where indirect exciton also exhibits prominent features due to carrier transfer between different valleys (such as K to Q valley). Nevertheless, several studies have reported a slight variation in the absorption spectrum of TMDs heterostructure due to strong interlayer coupling.[18–20] However, no indirect peak (Q-exciton) was observed unlike that in PL.[21]

We first measured absorption on the SiO_2 substrate (Supplementary Fig. S23a) to make a direct comparison with our PL data. We found that each peak is redshifted in WS_2/MoS_2 heterostructures compared to monolayer peaks, analogous to the redshift observed in bilayer compared to monolayer, as reported previously,[21–23] and this behavior has been attributed to the transition from a direct to an indirect bandgap. We note that no additional peak related to K-Q exciton was observed in this case and such an absence of K-Q peak from absorption may originate from indirect nature. Similar K-Q peak from absorption not appeared from quartz substrate (Supplementary Fig. S23b) as well, although K-Q exciton in PL was observed (Supplementary Fig. S14a). Therefore, we conclude that K-Q peaks cannot be observed from absorption, congruent with previous reports as well.[21]

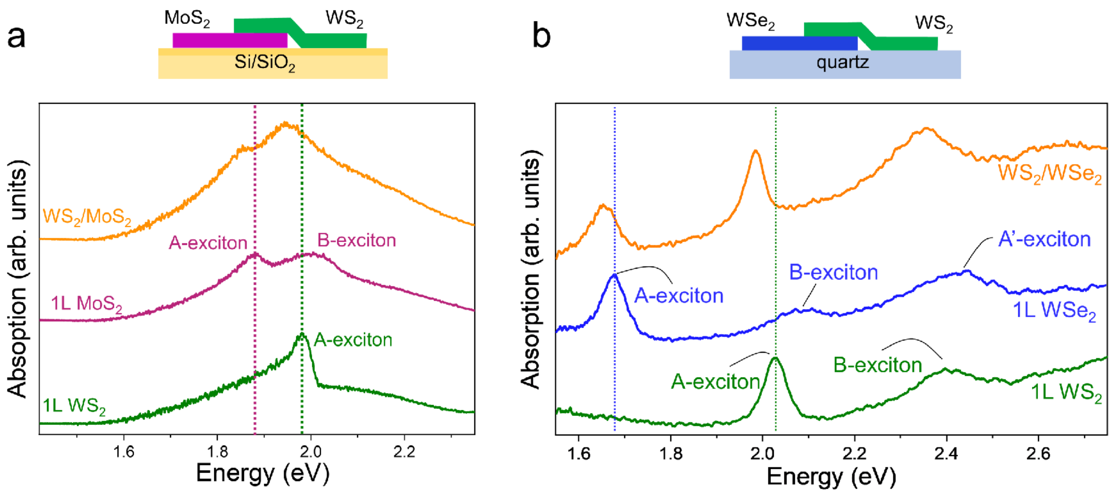

Fig. S23 | Absorption measurements. a, Absorption spectra of 1L WS_2, 1L MoS_2, and WS_2/MoS_2 heterostructure on SiO_2 substrate. **b,** Absorption spectra of 1L WS_2, 1L WSe_2, and WS_2/WSe_2 heterostructure on quartz substrate.

Note 16. Charge transfer and the related exciton dynamics

The charge transfer at the heterostructure is the key to explain the intensity modulation of K and Q-excitons. The PL intensity of neutral excitons of WSe_2 at heterostructure was greatly reduced compared to the individual WSe_2 monolayer region (Supplementary Fig. S24a) due to efficient charge transfer from WSe_2 to WS_2. Meanwhile, Q-exciton intensity was remarkably developed, whereas the intensity of WS_2 K-exciton was also reduced at the heterostructure due to further charge transfer from K to Q-band (Supplementary Fig. S24b). Supplementary Fig. S24c shows two relaxation times of fast relaxation τ_1 (WS_2: ~0.974 ns and WSe_2: ~1.406 ns) due to presumably exciton scattering or electron-phonon scattering[24] and slow relaxation time τ_2 (WS_2: ~2.624 ns and WSe_2: ~3.326 ns) due to trions and/or traps.[25] Such behaviors of two relaxation times of individual WSe_2 and WS_2 monolayers are similar to each other. Notably, additional faster relaxation (~0.587 ns) appears at the heterostructure. This can be explained again by the efficient charge transfer from WSe_2 to WS_2 and further from K to Q-band in WS_2. This is consistent with the previous report on faster relaxation time in multilayered WS_2 compared to monolayer.[26]

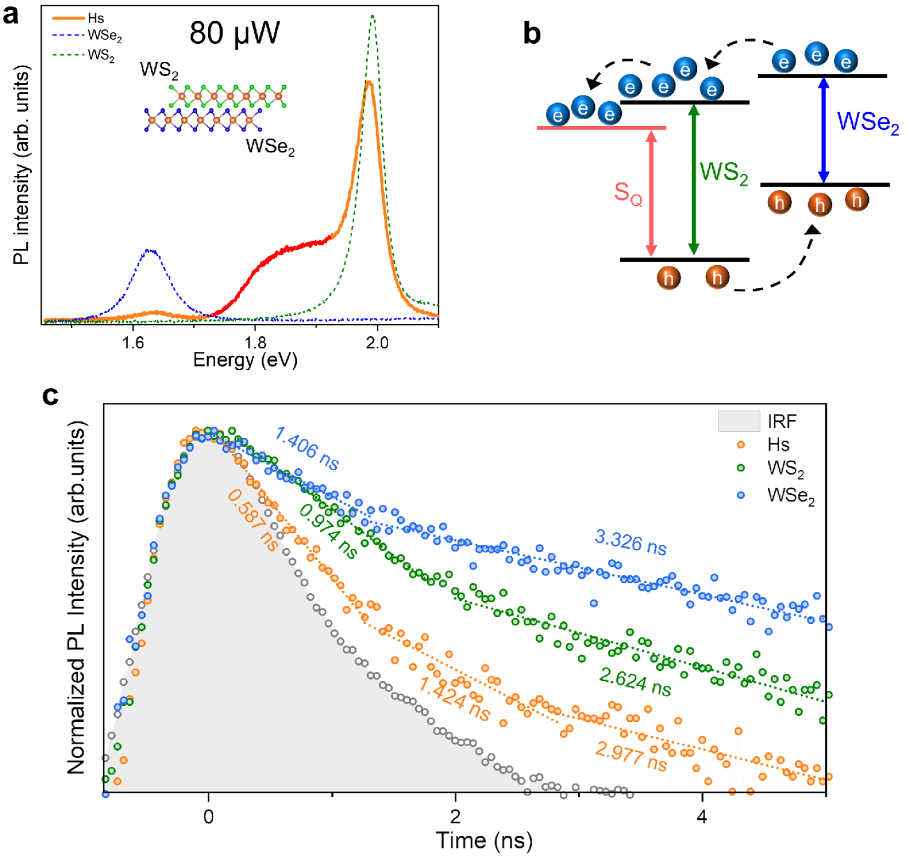

Fig. S24 | Charge transfer at heterostructure. a,b, PL measured at individual monolayers (WSe_2: blue-dotted line; WS_2: green-dotted line) as well as WS_2/WSe_2 heterostructure (yellow solid line) at the same excitation power. PL intensity is drastically reduced on WSe_2 side compared to WS_2 at the heterostructure region, which indicates the majority of the charge transferred from WSe_2 to

K and Q-band of WS_2 as represented in **b**. **c,** TRPL measurement of monolayer WSe_2, WS_2 and WS_2/WSe_2 heterostructure.

In this report, we focused solely on the K-K and K-Q excitons in the heterostructure. To accomplish this, we need to perform simultaneous measurements of each K-K and K-Q peak separately and their corresponding relaxation time. However, simultaneous measurements of such discrete peaks cannot be possible, primarily due to their close proximity (as wavelength less equal to 50 nm). As a result, performing such precise measurements are beyond the scope of the current work and requires further investigations.

Note 17. Mechanical cleaning *via* AFM tip

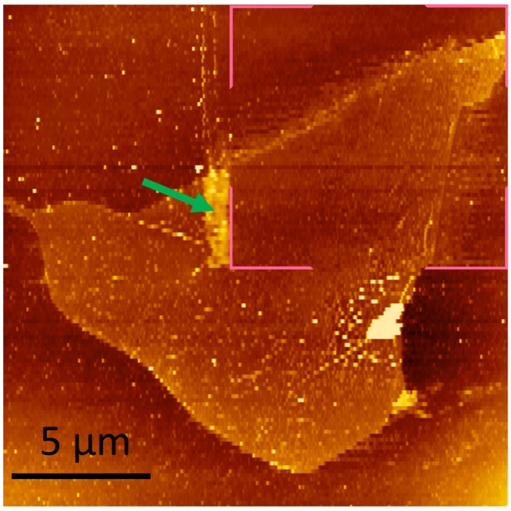

Fig. S25 | Mechanical cleaning by contact mode AFM. After contact mode scanning a tapping mode image has been taken to check the surface cleanness. The pink marked box region indicates the cleaned area and the wall of collected residue is indicated by an arrow on the left side of the box.

Note 18. Scanning tunneling spectroscopy (STS) measurement

From Fig. 1 we observed that the energy difference between S_Q peak and WS_2 A-exciton is less than 100 meV, which implies that the thermal broadening of room temperature STS measurement will heavily interfere in resolving such a small energy gap.[27] However, 2L-WS_2/1L-WSe_2 heterostructure provides an S_Q peak (S_{Q2} peak in Supplementary Fig. S19c) that is greater than 100 meV gap from the nearest A-exciton peak. Thus, we fabricated our heterostructure in such a way that it consists of a few regions 2L-WS_2/1L-WSe_2 and the rest of the 1L-WS_2/1L-WSe_2 area to ensure a certain signature of S_Q peak from room temperature STS measurement. The additional density of states near the conduction band edge (red peak) was clearly visible at the 2L-WS_2/1L-WSe_2 heterostructure region (Supplementary Fig. S26).

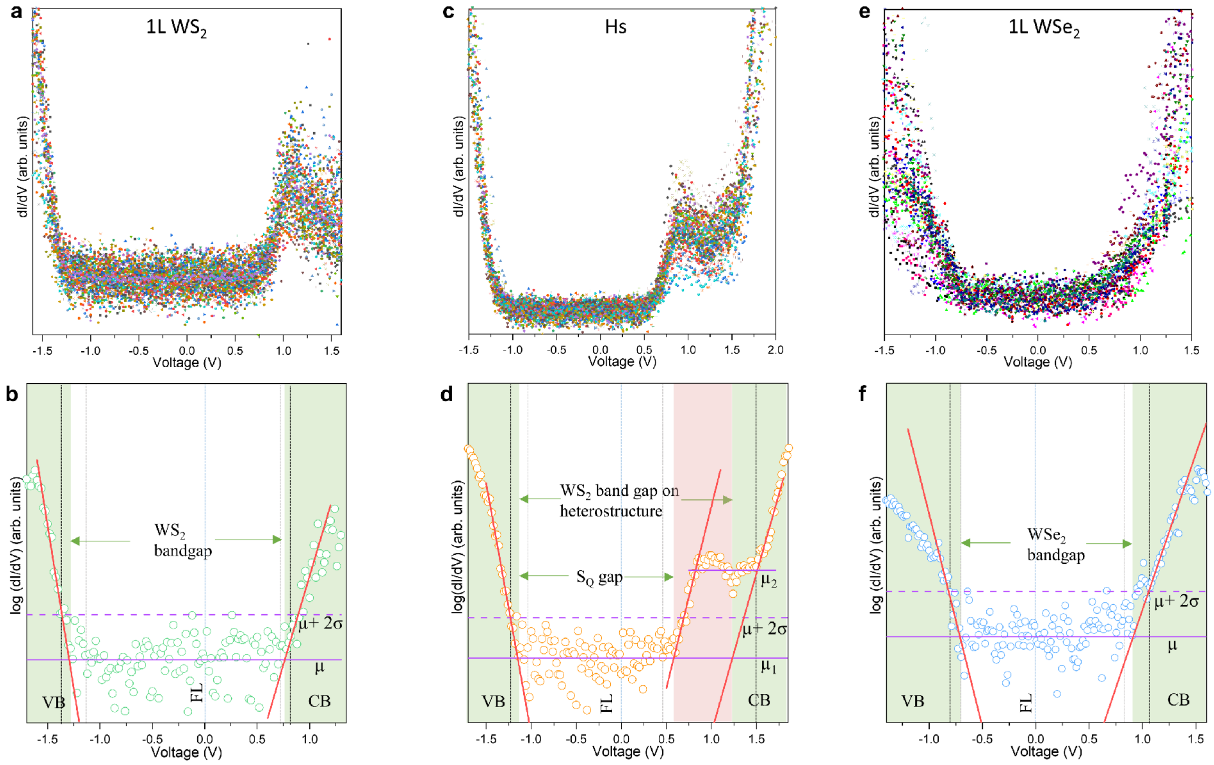

Fig. S26 | STS measurements. a-f, STS was measured at monolayer WS_2, 2L-WS_2/1L-WSe_2 heterostructure, and monolayer WSe_2 region respectively, and their corresponding bandgap. Bandgap has been calculated by average over individual 60 STS for WS_2 monolayer, 50 STS for heterostructure, and 35 STS for WSe_2 monolayer region.

Note 19. Laser power-dependent measurement of WS_2/WSe_2 on SiO_2

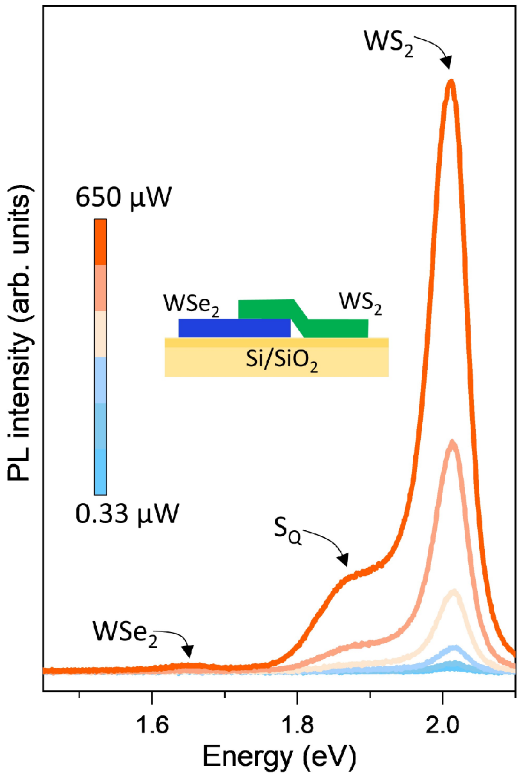

Fig. S27 | Power-dependent PL measurement. Power-dependent PL measurement at WS_2/WSe_2 heterostructure on SiO_2 substrate.

Note 20. S_Q intensity mapping

PL intensity mapping may give a more detailed view of S_Q peak distribution in real space. As shown in Supplementary Fig. 28a, the integrated PL intensity of WS_2/WSe_2 heterostructure is plotted. Since the PL intensity of WS_2 is quite high compared to WSe_2, the WS_2 PL intensity is dominant compared to the heterostructure as well as the WSe_2 region. To alleviate this difficulty, we normalized the S_Q intensity with respect to WS_2 PL intensity and plotted it in Supplementary Fig. 28b. This gives the distribution of the S_Q peak over the heterostructure region, where the S_Q population is well represented compared to the intrinsic inlayer WS_2 intensity. The S_Q intensity is dominant at the heterostructure region as shown in Supplementary Fig. 28b (yellow to the red region: scale >1). Furthermore, the intensity ratio changes from position to position, as described in Fig. 4c due to the variation of coupling strength between the layers.

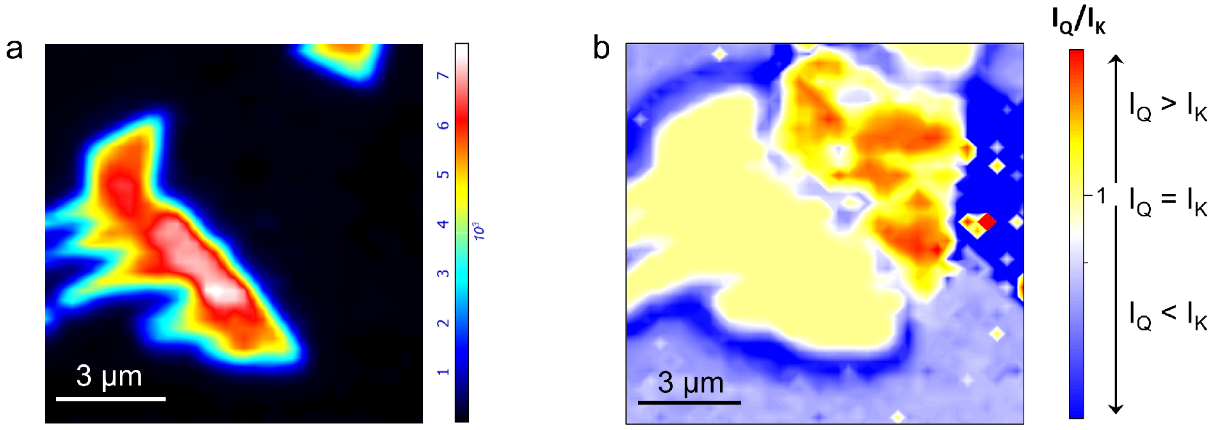

Fig. S28 | Intensity mapping. a, Integrated PL intensity of WS_2/WSe_2 heterostructure, where WS_2 PL intensity is dominant compared to the heterostructure as well as the WSe_2 region. **b,** Normalized S_Q intensity with respect to WS_2 PL intensity.

Note 21. Raman spectra at different positions

We investigated Raman spectra to gain the nature of the possible interlayer strain effect by monitoring the $E^1{}_{2g}$ Raman modes for both stacking sequences. In our scenario, we mainly focused on the interlayer coupling-induced strain effect (without application of external strain), which can alter the vibration of the phonon modes in the heterostructure compared to the monolayer region. However, we found that the in-plane $E^1{}_{2g}$ modes of the heterobilayer show strong position-dependent phonon shifts (Supplementary Fig. S29), like - blue shift (compressive strain) or redshift (tensile strain) depending on the position. This result suggests complicated inhomogeneous local strain profiles that vary with positions.

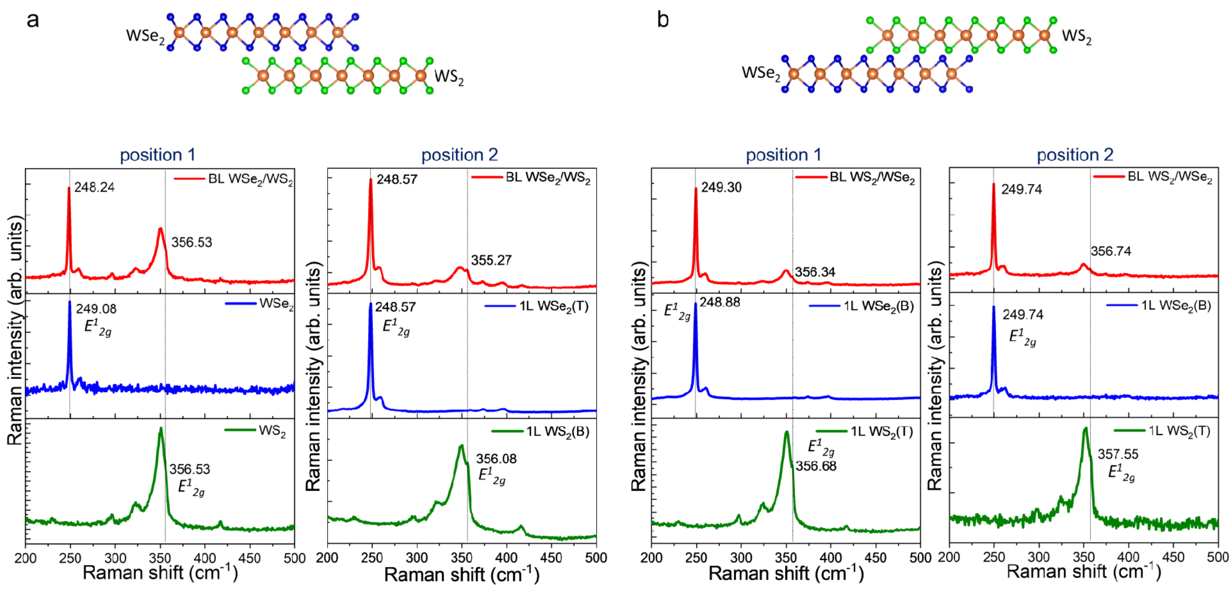

Fig. S29 | Raman spectra with different stacking sequences. a,b, Raman spectra in WSe_2/WS_2 and WS_2/WSe_2 heterostructure. Variation of $E^1{}_{2g}$ is noted for each layer along with heterostructure.

Note 22. Schematic representation of compressive force

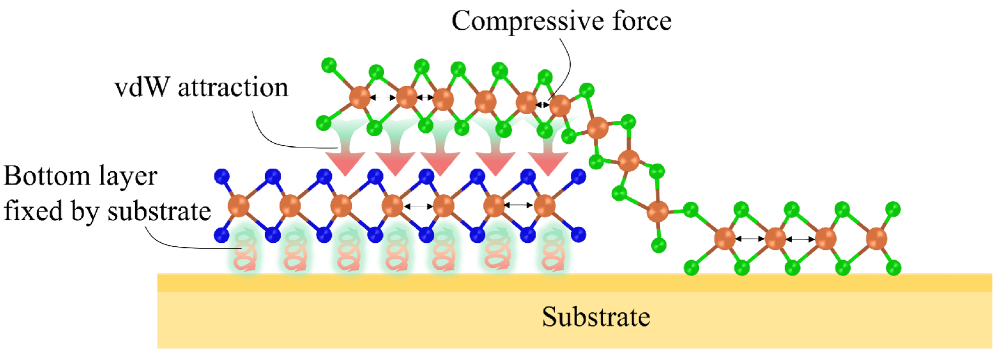

Fig. S30 | Strain on the top layer. Schematical representation that the bottom layer is fixed by the substrate whereas the top layer experiences a compressive strain.

Note 23. Theoretical calculations for comparing the K-Q to K-K excitons for W- and Mo-based materials.

The excitonic energies are microscopically evaluated starting from the unstrained single-particle dispersion[28] and including their strain-dependent variations[29]. These values are then used to numerically solve the Wannier equation by introducing a generalized Keldysh potential for the Coulomb interaction.

In Supplementary Fig. S31 top, we show the resulting excitonic center-of-mass dispersion of K-Q excitons for the WS_2 case, considering SiO_2/air as a dielectric environment. Already in the unstrained case (blue), the minimum K-Q energy is smaller than the bright-exciton energy $E_{K\text{-}K}$. In the presence of a compressive strain s=-0.30%, the energy separation increases by a factor of 3 from 30 meV up to almost 87 meV. In contrast, in $MoSe_2$ the K-Q valley is energetically above the K-K valley as shown in Supplementary Fig. S31 bottom. In this case, compressive strain also leads to a blueshift of K-Q energies (redshift of $E_{K\text{-}K}$). This reduces the energy separation between the K-Q and K-K excitons from 123 to 69 meV, while keeping K-K as the ground state.

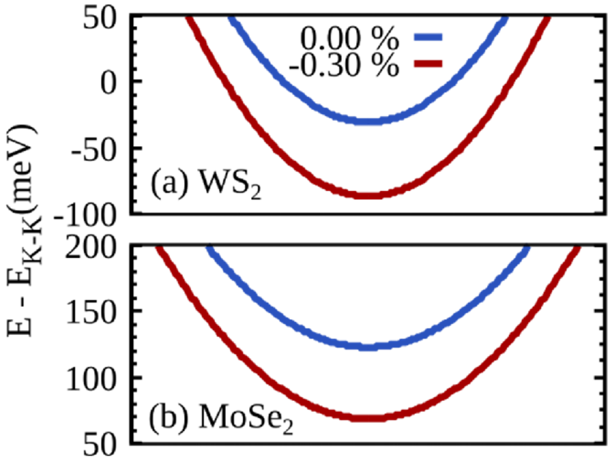

Fig. S31 | Dispersion of dark excitons. Energy of K-Q excitons as a function of the center-of-mass momentum for unstrained (blue) and compressively strained (red) (a) WS_2 and (b) $MoSe_2$.

In Supplementary Fig. S32, we provide a theory-experiment comparison for the WS_2-$MoSe_2$ heterostructure (Fig. 4e-f for WS_2-WSe_2). In the presence of a strained top layer, we find a new peak in WS_2/$MoSe_2$ in accordance with the experimentally measured S_Q, reflecting K-Q states with energy smaller than $E_{K\text{-}K}$ (Supplementary Fig. S31 top). In contrast, we find no new phonon-assisted peaks in $MoSe_2$/WS_2, as here the K-Q excitons are energetically above the bright ones (Supplementary Fig. S31 bottom). This implies their reduced occupation in comparison to the bright-exciton states, resulting in negligible phonon-assisted photoluminescence. Nevertheless, our prediction of a decreased $E_{K\text{-}Q}$-$E_{K\text{-}K}$ separation with compressive strain (Supplementary Fig.

S31 bottom) could potentially lead to a peak similar to Se_Q in $MoSe_2/WS_2$, if an additional activation mechanism is present, e.g. via interplay of strain and defects.[30] The microscopic evaluation of these mechanisms goes beyond the scope of this work.

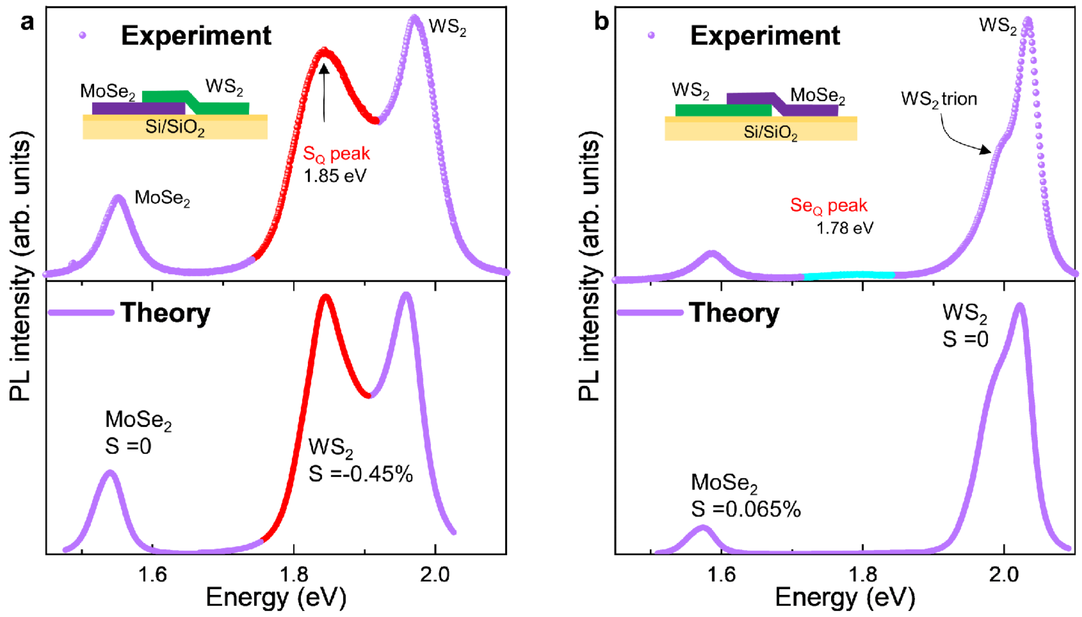

Fig. S32 | Experimental and theoretical PL for $MoSe_2$-WS_2 heterostructure. a, In the case of top-WS_2, a low-energy peak (S_Q) stems from the phonon-assisted transition by assuming a 0.45 % compressive strain in the WS_2 layer. **b,** In the case of the top-$MoSe_2$ layer, we do not see a phonon sideband on the high-energy side of the bright $MoSe_2$ exciton, as the occupation of the K-Q state is negligibly small.

Note 24. Independent of optical excitation path

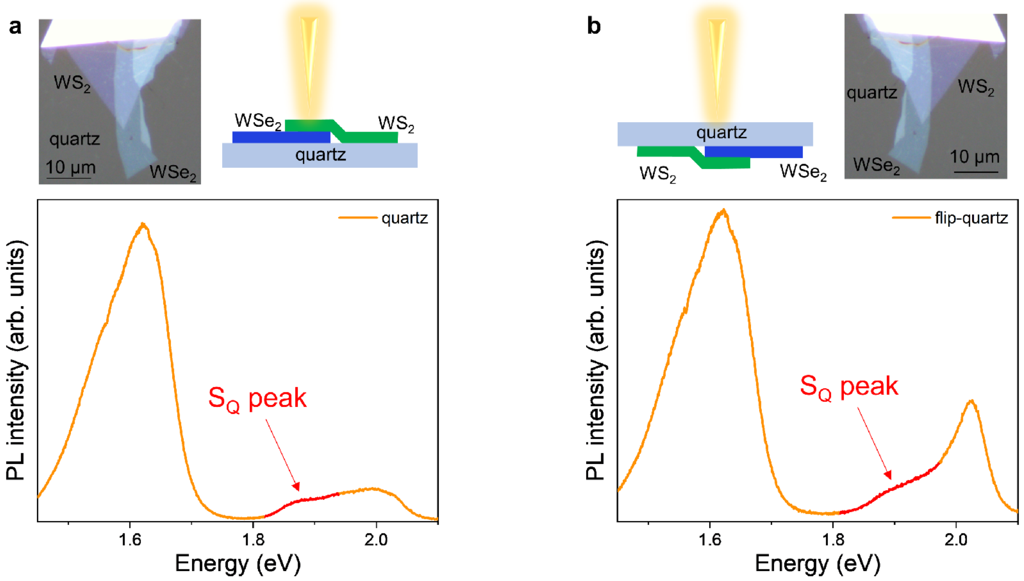

Fig. S33 | Optical path-dependent PL measurements with transparent quartz substrate. a,b, PL spectra of WS_2/WSe_2 heterostructure by illuminating the laser through the top and bottom of the heterostructure. The presence of S_Q peak in both cases indicates this phenomenon is independent of the optical path excitation.

References.

1. Chiu M -H *et al* Spectroscopic Signatures for Interlayer Coupling in MoS_2-WSe_2 van der Waals Stacking. *ACS Nano* 9649–9656 (2014).
2. Berkdemir, A. *et al.* Identification of individual and few layers of WS_2 using Raman Spectroscopy. *Sci. Rep.* **3**, 1–8 (2013).
3. Zeng, H. *et al.* Optical signature of symmetry variations and spin-valley coupling in atomically thin tungsten dichalcogenides. *Sci. Rep.* **3**, 4908–4916 (2013).
4. Rivera, P. *et al.* Observation of long-lived interlayer excitons in monolayer $MoSe_2$-WSe_2 heterostructures. *Nat. Commun.* **6**, 4–9 (2015).
5. Nagler, P. *et al.* Interlayer Excitons in Transition-Metal Dichalcogenide Heterobilayers. *Phys. Status Solidi Basic Res.* **256**, (2019).
6. Förg, M. *et al.* Moiré excitons in $MoSe_2$-WSe_2 heterobilayers and heterotrilayers. *Nat. Commun.* **12**, 1–7 (2021).
7. Jin, C. *et al.* Observation of moiré excitons in WSe_2/WS_2 heterostructure superlattices. *Nature* **567**, 76–80 (2019).
8. Sun, X. *et al.* Enhanced interactions of interlayer excitons in free-standing heterobilayers. *Nature* **610**, 478–484 (2022).
9. Yuan, L. *et al.* Twist-angle-dependent interlayer exciton diffusion in WS_2–WSe_2 heterobilayers. *Nat. Mater.* **19**, 617–623 (2020).
10. Zhang, L. *et al.* Twist-angle dependence of moiré excitons in WS_2/$MoSe_2$ heterobilayers. *Nat. Commun.* **11**, 1–8 (2020).
11. Nayak, P. K. *et al.* Probing Evolution of Twist-Angle-Dependent Interlayer Excitons in $MoSe_2$/WSe_2 van der Waals Heterostructures. *ACS Nano* **11**, 4041–4050 (2017).
12. Ye, T., Li, J. & Li, D. Charge-Accumulation Effect in Transition Metal Dichalcogenide Heterobilayers. *Small* **15**, 1–6 (2019).
13. YU, J. *et al.* Observation of double indirect interlayer exciton in WSe_2/WS_2 heterostructure. *Opt. Express* **28**, 13260–13268 (2020).
14. Liu, F., Li, Q. & Zhu, X. Y. Direct determination of momentum-resolved electron transfer in the photoexcited van der Waals heterobilayer WS_2/MoS_2. *Phys. Rev. B* **101**, 201405 (2020).
15. Tongay, S. *et al.* Tuning Interlayer Coupling in Large-Area Heterostructures with CVD-Grown MoS_2 and WS_2 Monolayers, Supporting information. *Nano Lett.* **14**, 3185–3190 (2014).
16. Darlington, T. P. *et al.* Imaging strain-localized excitons in nanoscale bubbles of monolayer WSe_2 at room temperature. *Nat. Nanotechnol.* **15**, 854–860 (2020).
17. Sebait, R., Biswas, C., Song, B., Seo, C. & Lee, Y. H. Identifying defect-induced trion in

monolayer WS_2 via carrier screening engineering. *ACS Nano* **15**, 2849–2857 (2021).

18. Qin, C. *et al.* Observation of Hole Transfer in MoS_2/WS_2 Van der Waals Heterostructures. *ACS Photonics* (2022) doi:10.1021/acsphotonics.2c00078.

19. Wang, K. *et al.* Interlayer Coupling in Twisted WSe_2/WS_2 Bilayer Heterostructures Revealed by Optical Spectroscopy. *ACS Nano* **10**, 6612–6622 (2016).

20. Ansari, N. & Ghorbani, F. Light absorption optimization in two-dimensional transition metal dichalcogenide van der Waals heterostructures. *J. Opt. Soc. Am. B* **35**, 1179 (2018).

21. Zhao, W. *et al.* Evolution of electronic structure in atomically thin sheets of WS_2 and WSe_2. *ACS Nano* **7**, 791–797 (2013).

22. Niu, Y. *et al.* Thickness-dependent differential reflectance spectra of monolayer and few-layer MoS_2, $MoSe_2$, WS_2 and WSe_2. *Nanomaterials* **8**, (2018).

23. Gomez C. *et al.* Spatially resolved optical absorption spectroscopy of single- and few-layer MoS_2 by hyperspectral imaging. *Nanotechnology* **27**, (2016).

24. Tanoh, A. O. A. *et al.* Directed energy transfer from monolayer WS_2 to near-infrared emitting PbS-CDS quantum dots. *ACS Nano* **14**, 15374–15384 (2020).

25. Choi, C. *et al.* Enhanced interlayer neutral excitons and trions in trilayer van der Waals heterostructures. *npj 2D Mater. Appl.* **2**, (2018).

26. Yuan, L. & Huang, L. Exciton dynamics and annihilation in WS_2 2D semiconductors. *Nanoscale* **7**, 7402–7408 (2015).

27. C. Julian Chen. *Introduction to Scanning Tunneling Microscopy*. vol. 15 (2016).

28. Kormányos, A. *et al.* k.p Theory for Two-Dimensional Transition Metal Dichalcogenide Semiconductors . *2D Mater.* **2**, 022001 (2015).

29. Khatibi Z. F *et al.* Impact of strain on the excitonic linewidth in transition metal dichalcogenides. *2D Mater.* **6**, (2019).

30. Hernández López, P. *et al.* Strain control of hybridization between dark and localized excitons in a 2D semiconductor. *Nat. Commun.* **13**, 1–9 (2022).
